# β-adrenergic regulation of Ca^2+^ signaling in heart cells

**DOI:** 10.52601/bpr.2024.240906

**Published:** 2024-10-31

**Authors:** Bo Yang, Shi-Qiang Wang, Hua-Qian Yang

**Affiliations:** 1 Cyrus Tang Medical Institute, Jiangsu Key Laboratory of Preventive and Translational Medicine for Geriatric Diseases, Soochow University, Suzhou, Jiangsu 215123, China; 2 State Key Lab of Membrane Biology, College of Life Sciences, Peking University, Beijing 100871, China

**Keywords:** β-adrenergic receptor (βAR), Ca^2+^ signaling, Cardiac myocytes, Compartmentalization

## Abstract

β-adrenergic receptors (βARs) play significant roles in regulating Ca^2+^ signaling in cardiac myocytes, thus holding a key function in modulating heart performance. βARs regulate the influx of extracellular Ca^2+^ and the release and uptake of Ca^2+^ from the sarcoplasmic reticulum (SR) by activating key components such as L-type calcium channels (LTCCs), ryanodine receptors (RyRs) and phospholamban (PLN), mediated by the phosphorylation actions by protein kinase A (PKA). In cardiac myocytes, the presence of β_2_AR provides a protective mechanism against potential overstimulation of β_1_AR, which may aid in the restoration of cardiac dysfunctions. Understanding the Ca^2+^ regulatory signaling pathways of βARs in cardiac myocytes and the differences among various βAR subtypes are crucial in cardiology and hold great potential for developing treatments for heart diseases.

## INTRODUCTION

β-adrenergic receptors (βARs) belong to the G protein-coupled receptor (GPCR) superfamily, and are essential for regulating the function of the cardiovascular system. βARs are activated by catecholamines released from sympathetic nerve terminals and adrenal medulla under stress conditions, which increase heart rate and blood pumping capability of the heart (Bers 2002). The positive chronotropic, dromotropic and inotropic effects ensure the energy supply of emergent needs.

Currently, there are three identified subtypes of βARs (β_1_AR, β_2_AR, β_3_AR), while the existence of a fourth subtype (β_4_AR) is still a subject of debate (Gauthier *et al.* 1996). These three isoforms exhibit different affinities for different ligands, rendering the selectivity of isoform activation (Bristow *et al.* 1986). The ratio of β_1_AR/β_2_AR expression in the healthy human heart is approximately 4:1, while the expression of β_3_AR is minimal. Both β_1_AR and β_2_AR respond to catecholamine stimulation and mediate positive inotropic effects in heart cells. β_1_AR plays a dominant role in increasing chronotropy and inotropy in cardiac myocytes, whereas β_2_AR produces only modest chronotropic effects (Xiang and Kobilka 2003; Xiao *et al.* 2006).

**Figure 1:**
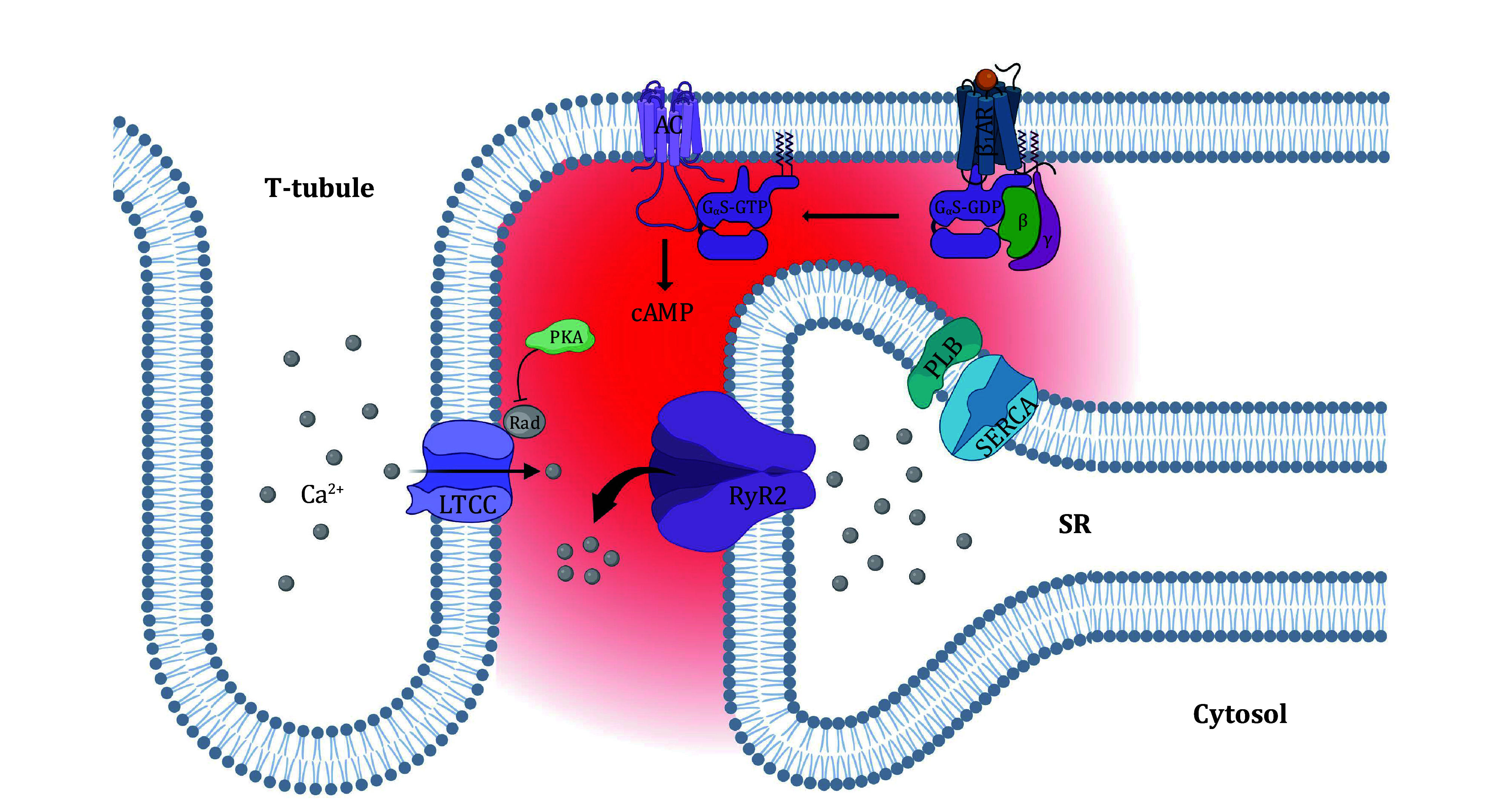
Illustration of β-adrenergic regulation of Ca^2+^ signaling in heart cells

Epinephrine and norepinephrine are native catecholamine ligands of βARs (Bunemann *et al.* 1999; Hain *et al.* 1995; Nikolaev *et al.* 2006; Nikolaev *et al.* 2010). With catecholamine binding, βARs undergo conformational changes that enable its coupling to heterotrimeric G proteins, resulting in the substitution of the GDP on the G_α_ subunit of G-proteins by GTP and subsequent dissociation of G_βγ_ subunits (Wess 1997). G_α_-GTP then stimulates adenylyl cyclase (AC) to catalyze the formation of cyclic AMP (cAMP). cAMP regulates a wide variety of cellular processes through activating a variety of downstream signaling molecules, including protein kinase A (PKA). PKA phosphorylates L-type Ca^2+^ channels (LTCCs) in the cell membrane or T-tubules, ryanodine receptor (RyR) Ca^2+^ release channels and phospholamban (PLN) in the sarcoplasmic reticulum (SR), and thereby up-regulates LTCC Ca^2+^ influx, SR Ca^2+^ release and cytosolic Ca^2+^ uptake (Fig. 1).

## REGULATION OF LTCC

LTCCs are the predominant mediator of Ca^2+^ influx in the cardiomyocytes playing an initiation role in the excitation-contraction coupling. In general, LTCCs are composed of α_1_, α_2_, β, δ and γ subunits. α_1_ is the pore-forming subunit with voltage sensors. α_2_/δ and β subunits modulate the expression, voltage dependence and gating kinetics of the channel (Bodi *et al.* 2005).

Several PKA phosphorylation sites in the α_1_ subunit have been identified (Fu *et al.* 2014; Hulme *et al.* 2006; Yang *et al.* 2016). βAR agonists, such as isoproterenol, increase the phosphorylation level of S1928 in the distal C-terminal domain (Hulme *et al.* 2006), which can be blocked by βAR antagonists. Interestingly, LTCCs with S1928 mutated to alanine still retain 70%–80% response to βAR stimulation, indicating that S1928 is not the major phosphorylation site for βAR stimulation (Benitah *et al.* 2010; Ganesan *et al.* 2006; Hulme *et al.* 2003, 2006). S1700 and T1704 located at the interface between the proximal and distal C-terminal domain are also phosphorylated in βAR regulation of LTCCs (Fu *et al.* 2013, 2014). Again, mutations of both S1700 and T1704 cannot eliminate βAR effects (Fu *et al.* 2013).

The regulatory β_2_ subunit plays a crucial role in LTCCs regulation in response to βAR stimulation (Haase *et al.* 1993). S478 and S479 were identified as the phosphorylation sites of PKA in β_2_ subunit (Gerhardstein *et al.* 1999). Mutating S478 and S479 to alanine in the β_2_ subunit inhibits PKA-mediated Ca^2+^ current increase in transfected cells (Bunemann *et al.* 1999). This result suggests that phosphorylation of S478 or S479 contributes to PKA-mediated regulation of LTCCs.

Monomeric G proteins, such as Rem and Rad, function as endogenous LTCC inhibitors (Beguin *et al.* 2001; Finlin *et al.* 2003). Recent analysis from a proximity proteomics screen provided solid evidence that Rad is enriched in the LTCC microenvironment but is depleted during β-adrenergic stimulation. Phosphorylation by PKA decreases Rad affinity for β subunits and increases LTCC open probability (Liu *et al.* 2020). Four serines in Rad have been identified as PKA phosphorylation sites, and mutation of these four serines or disrupting the interaction between LTCC β subunit and Rad reduced heart rate and basal contractility, and greatly diminished β-adrenergic contractile response (Papa *et al.* 2022, 2024).

A kinase anchoring protein 15 (AKAP15) is a lipid-anchored protein with a single amphipathic helix that binds PKA. AKAP15 colocalizes and associates with LTCC in T-tubules (Gray *et al.* 1998). PKA tethered to a leucine zipper motif in the C-terminal domain of the LTCC α_1_ subunit via AKAP15 (Hulme *et al.* 2002), which is essential for β-adrenergic regulation of LTCC (Hulme *et al.* 2003).

LTCCs are also phosphorylated by Ca^2+^/calmodulin-dependent kinase II (CaMKII). CaMKII is activated by βAR stimulation via guanine nucleotide exchange protein directly activated by cAMP (Epac) (Curran *et al.* 2007; Grimm and Brown 2010). Mutations at sites S1512 and S1570 of α_1_ subunit (Hudmon *et al.* 2005) and T498 of β2 subunit (Koval *et al.* 2010) reduce Ca^2+^ influx.

Besides, cardiac phosphatase activities also play important roles in the regulation of Ca^2+^ homeostasis. Phosphatase type 1 (PP1) and 2A (PP2A) are the major isotypes of cardiac phosphatases, comprising over 90% of the protein phosphatases in cardiomyocytes (Lüss *et al.* 2000). PP1 is reported to contribute to the dephosphorylation of LTCC, RyR, and PLB. Whereas, PP2A is mainly involved in the dephosphorylation of myofibrillar proteins, including troponin I and myosin-binding protein C (Metzger and Westfall 2004).

In recent years, a few proteins have been reported to modify the β-adrenergic regulation of LTCCs. Sphingosine-1-phosphate (S1P), a circulating bioactive sphingolipid, has been implicated in the regulation of several cellular processes including cardiac Ca^2+^ handling (Means and Brown 2009). S1P does not affect the basal LTCC current, but partially reverses the regulation of βAR activation on LTCCs through a signaling pathway involving the interaction between P21-activated kinase 1 (Pak1) and protein phosphatase 2A (PP2A) (Egom *et al.* 2016). Ahnak functions as a suppressor of LTCCs by sequestering the β_2_ subunit through a strong binding to the LTCC β_2_ subunit (Hohaus *et al.* 2002). Rem GTPase interacts with LTCC β_2_ subunit and inhibits LTCC currents. The inhibitor effects can be rescued by LTCC activators such as BayK8644, but not by the βAR stimulation (Xu *et al.* 2010). Besides the functional coupling regulators, there were some structural coupling factors, such as Bridging Integrator 1 (BIN1) and caveolin-3. BIN1 is essential for the localization of LTCCs to T-tubules in cardiomyocytes and affects LTCC regulation by βAR stimulation (Kumari *et al.* 2018). In heart cells, a subpopulation of LTCCs localizes in caveolae. Caveolae are specialized membrane microdomains and are supported by the structural protein caveolin-3. It is well known that β_2_AR is enriched in caveolae. There is evidence showing that regulation of LTCCs by β_2_AR, but not β_1_AR, is eliminated when caveolae were disrupted (Balijepalli *et al.* 2006). This indicates that LTCCs are coupled to β_1_AR signaling outside of caveolae.

## REGULATION OF RyRs

RyRs are major Ca^2+^ release channels in the SR of striated myocytes or endoplasmic reticulum (ER) of other cells. RyRs bind to ryanodine in their open state. Early studies using radiolabeled ryanodine have shown that phosphorylation of RyR2 by PKA increased channel activity (Takasago *et al.* 1991). However, the identification of the phosphorylation site critical for βAR response has been highly controversial. It has been proposed that the phosphorylation of S2808 by PKA sensitizes the response of RyRs to cytosolic Ca^2+^ change (Wehrens *et al.* 2004a). However, the mouse model harboring the S2808A mutation has normal inotropic and chronotropic responses to βAR stimulation (MacDonnell *et al.* 2008). S2808A cardiomyocytes exhibit blunted enhancement of systolic Ca^2+^ transients at 3 Hz but not at lower frequencies (Benkusky *et al.* 2007). There is also evidence that the phosphorylation of S2030 by PKA enhances RyR2 responsiveness to luminal Ca^2+^ (Xiao *et al.* 2005, 2007). However, data from different labs questioned Ser2030 as a physiological PKA phosphorylation site (Huke and Bers 2008; Wehrens *et al.* 2006).

Besides PKA-mediated phosphorylation, βAR-activated CaMKII specifically phosphorylates S2815 in RyRs (Kushnir *et al.* 2010; Wehrens *et al.* 2004b). Phosphorylation at S2815 increases the open probability of RyR2 by sensitizing the channel (Wehrens *et al.* 2004b). While cardiac-specific CaMKII overexpression enhances SR Ca^2+^ fractional release (Maier *et al.* 2003), cardiac-specific inhibition of CaMKII reduces isoproterenol-induced responses in SR Ca^2+^ release and heart rate (Wu *et al.* 2009).

In intact cells, βAR modulation of RyR function is difficult to measure, because βAR also increases LTCC Ca^2+^ current and SR Ca^2+^ loading. With a high-affinity Ca^2+^ indicator combined with a slow Ca^2+^ buffer agent EGTA to elicit Ca^2+^ spikes, it is demonstrated that isoproterenol synchronizes the Ca^2+^ release from RyR clusters (Song *et al.* 2001). When the SR Ca^2+^ load and Ca^2+^ current were controlled, isoproterenol stimulation of β_1_AR accelerates SR Ca^2+^ release kinetics without altering the amplitude of Ca^2+^ transients (Ginsburg and Bers 2004), agreeing well with the PKA-mediated synchronization of RyR Ca^2+^ release (Lakatta 2004; Wang and Wehrens 2010). However, experiments using UV photolysis to activate RyRs showed that isoproterenol enhances both the speed and the magnitude of Ca^2+^ transients in cells with controlled SR Ca^2+^ load (Ogrodnik and Niggli 2010). Using the loose-sealed patch clamp to trigger individual RyR Ca^2+^ release units, manifested as a Ca^2+^ spark, we observed that selective βAR stimulation enhances the amplitude of triggered sparks in an LTCC unitary current-independent manner. The Ca^2+^ release flux that underlies a Ca^2+^ spark is enhanced when the SR Ca^2+^ content is controlled to a comparable level. These results demonstrate unequivocally that the activation of RyRs is expedited and synchronized under βAR stimulation (Zhou *et al.* 2009).

## REGULATION OF PLN

The rapid removal of Ca^2+^ from the cytoplasm is primarily facilitated by the sarco(endo)plasmic reticulum Ca^2+^ ATPase SERCA2a, which pumps Ca^2+^ back into the SR cavity and thus controls the amount of Ca^2+^ in the SR (Zhihao *et al.* 2020). PLB is the endogenous regulatory protein of SERCA2a activity and is the only regulatory protein of SERCA2a that is directly involved in the development of heart disease, including heart failure (Shanmugam *et al.* 2011; Weber *et al.* 2021).

There are two phosphorylation sites in PLN, Ser16 and Thr17, which are phosphorylated by PKA and CaMKII respectively (Kuschel *et al.* 1999; Simmerman *et al.* 1986; Xiao *et al.* 1994). Experiments with phosphorylation of PLN at either site increase SR Ca^2+^ load, and thus enhance SR Ca^2+^ release and accelerate cardiomyocyte relaxation (Li *et al.* 2002). Different from that of Ser16, the phosphorylation of Thr17 by β_1_AR is enhanced with increased frequency of electrical stimulation possibly because frequency-dependent accumulation of intracellular Ca^2+^ facilities CaMKII activation (Hagemann *et al.* 2000).

## DIFFERENCE BETWEEN β_1_AR AND β_2_AR SIGNALING

**Figure 2:**
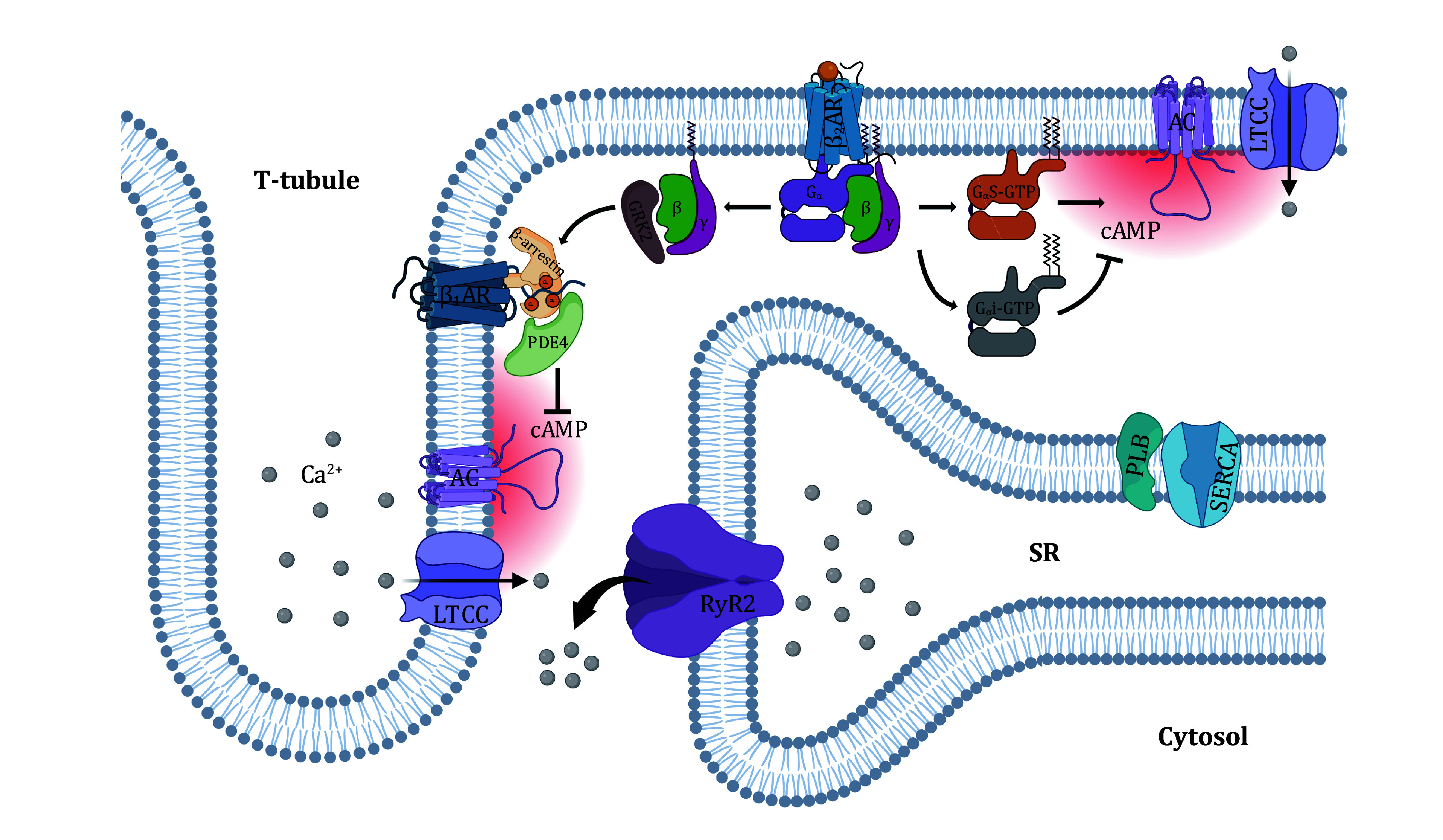
Illustration of compartmentalized β_2_AR-cAMP signaling in heart cells

The amino acid sequences of human β_1_AR and β_2_AR share only 71% identity in the transmembrane domains and 54% identity overall (Dixon *et al.* 1986). In the heart, β_1_AR-activated cAMP signaling increases the phosphorylation of sarcolemmal LTCCs and a multitude of intracellular regulatory proteins, including RyR, PLB and myofilaments (Xiao 2001). However, β_2_AR-mediated cAMP signaling specifically modulates LTCCs without affecting PLB and myofilaments in most mammalian species (Fig. 2) (Xiao and Lakatta 1993). Although in the human heart, β_2_AR stimulation increases PKA-dependent phosphorylation of intracellular regulatory proteins, its effects are much smaller than that induced by β_1_AR stimulation (Altschuld *et al.* 1995). Furthermore, β_2_ARs are expressed preferentially in the T-Tubule membrane, while β_1_ARs are distributed in both T-tubules and surface membrane (Nikolaev *et al.* 2010).

βARs are G protein-coupled receptors. β_1_AR and β_2_AR both couple to G_s_ protein, while β_2_AR also couples to G_i_ protein (Fig. 2). Selective β_2_AR stimulation by zinterol does not enhance cardiomyocyte contraction in both wild-type (WT) mice and transgenic mice overexpressing human β_2_AR (TG4) (Zhou *et al.* 1999). After incubating cells with pertussis toxin (PTX), which abrogates G_i_/G_o_ function via ADP ribosylation, zinterol markedly increases contraction amplitude in both WT and TG4 cardiomyocytes, which can be completely abolished by the specific β_2_AR antagonist (Xiao *et al.* 1999). In cell-attached patch clamp experiment, β_2_AR agonist in bath solution outside the patch pipette cannot cause a discernible change in LTCC activity in the patch membrane, while local β_2_AR agonist in the pipette markedly increases the open probability of the patched channel. This sophisticated experiment indicates that β_2_AR signaling is confined in a highly localized microdomain. After PTX treatment, the channels in the patch membrane became responsive to agonist in the bath solution, suggesting that G_i_ plays an essential role in the compartmentalized β_2_AR signaling (Chen-Izu *et al.* 2000).

In addition to the impact of G_i_ protein, it is proposed that β_2_ARs reside in caveolae, which compartmentalize β_2_AR signaling. Indeed, caveolin-3 is of vital importance for the localization of β_2_AR and compartmentation of β_2_AR-cAMP signaling in healthy cardiomyocytes (Wright *et al.* 2014). Also, phosphodiesterase 4D (PDE4D) is recruited by β-arrestin2 to the vicinity of β_2_AR. Its hydrolysis of cAMP restricts the spatial diffusion of β_2_AR-activated cAMP signal (Fischmeister *et al.* 2006; Richter *et al.* 2008; Shi *et al.* 2017). Endogenous catecholamine ligands of βARs, epinephrine and norepinephrine, induced distinct β_2_AR signaling through G protein-coupled receptor kinase 2 (GRK2) phosphorylation and selective binding of G_s_ or G_i_ (Heubach *et al.* 2004; Wang *et al.* 2008), which further revealed the complexity of β_2_AR downstream signaling.

## β_2_AR-MEDIATED OFFSIDE COMPARTMENTALIZATION OF β_1_AR SIGNALING

Accumulative evidence suggests that β_1_AR and β_2_AR pathways may have crosstalk. The activation of β_2_AR has been found to blunt the signaling of β_1_AR in failing heart cells (He *et al.* 2005). In transgenic mice overexpressing β_2_ARs, the contractility of cardiomyocytes is enhanced through spontaneous β_2_AR-cAMP signaling. However, these cells lose their ability to respond to β_1_AR stimulation (Zhang *et al.* 2000).

Recently, we have analyzed the interaction between β_2_AR and β_1_AR signaling. While isoproterenol normally up-regulates Ca^2+^ transients during cardiomyocyte excitation, salbutamol, a selective β_2_AR agonist, hinders the ability of isoproterenol to regulate Ca^2+^ transients (Yang *et al.* 2019). This effect can be eliminated either by rolipram, a PDE4 inhibitor, or by peptides that antagonize β-arrestin1. In the rat model harboring mutations of the phosphorylation sites in the C-terminus of β_1_AR, a putative binding domain for β-arrestin1 and GRK2, β_2_AR agonist no longer interferes with β_1_AR signaling. This study suggests that β_2_AR stimulation activates GRK2 to phosphorylate the C-terminus of β_1_AR, facilitates the recruitment of PDE4 to the phosphorylated β_1_AR, and compartmentalizes β_1_AR-cAMP signals within a sub-membrane nanodomain, preventing the PKA-dependent regulation of RyR and PLB. Because the compartmentalization of the β_1_AR pathway is rendered by the β_2_AR pathway in an offside manner, this signaling process is described as “offside compartmentalization” (Fig. 2) (Yang *et al.* 2019).

It is important to mention that the activation of offside compartmentalization can occur *in vivo* through the use of epinephrine, which hinders the regulation of heart contraction by norepinephrine (Yang *et al.* 2020). Epinephrine exhibits a limited preference for β_2_AR over β_1_AR as an adrenal hormone (Baker 2010), while norepinephrine predominantly stimulates β- and α_1_ARs as a sympathetic neurotransmitter (Minneman *et al.* 1981) and exhibits selectivity for β_1_AR over β_2_AR due to different entrance pathways to the extracellular binding pockets (Xu *et al.* 2021). Epinephrine and a less quantity of norepinephrine are tonically released from the adrenal glands (Paur *et al.* 2012). As prolonged activation of β_1_AR leads to cytotoxicity (Wu *et al.* 2017; Zhu *et al.* 2003), the offside compartmentalization initiated by β_2_AR signaling can serve as a negative feed-forward mechanism preventing the tonic β_1_AR activation by circulating catecholamines. Under the offside compartmentalization, βAR signaling is still able to synchronize SR Ca^2+^ release by up-regulating LTCC Ca^2+^ influx (Yang *et al.* 2020) and enhance the transient response of β_1_AR to norepinephrine during sympathetic excitation. Hence, in contrast to the robust and predictable E-C coupling regulation through overall β_1_AR signaling, the compartmentalized βAR regulation of E-C coupling, while being moderate, exhibits an "autoadaptive" nature in response to various physiological and pathological circumstances.

## PATHOLOGICAL IMPLICATIONS

While βARs play essential roles in the physiological operation of Ca^2+^ signaling, their malfunction is implicated in a variety of pathological processes. Prolonged β_1_AR stimulation induces apoptosis in a CaMKII-dependent manner, and β_2_AR blockade exaggerates β_1_AR-induced apoptosis (Communal *et al.* 1999) possibly due to the absence of offside compartmentalization. In contrast, stimulation of β_2_AR protects cardiac myocytes against a wide range of apoptotic insults, including enhanced β_1_AR signaling, hypoxic treatment or induction of reactive oxygen species (ROS) (Zhu *et al.* 2001). Inhibition of β_2_AR-activated G_i_–G_βγ_–PI3K–PKB signaling eliminates these protective effects, and transforms β_2_AR signaling from anti-apoptotic to pro-apoptotic (Zhu *et al.* 2001; Chesley *et al.* 2000). Emerging evidence suggests that mitogen-activated protein kinase (MAPK) and extracellular signal-regulated protein kinases (ERK1 and ERK2) are also involved in β_2_AR-mediated anti-apoptotic signaling (Shizukuda and Buttrick 2002).

During the early-stage development of heart failure, the sympathetic nervous system adjusts its activity to increase cardiac output to compensate for the alterations of cardiac and peripheral hemodynamics (Toschi-Dias *et al.* 2017). However, the continuous hemodynamic stress promotes the chronic release of catecholamines. The elevated level of catecholamine (Bristow *et al.* 1982; Ungerer *et al.* 1993) leads to sustained and toxic β_1_AR-CaMKII signaling, which exacerbates the decline in cardiac function as observed in mid- and late-stages of heart failure (Brede *et al.* 2002; Johnson and Antoons 2018; Zhu *et al.* 2003).

## CONCLUDING REMARKS

Heart disease is the leading cause of death globally. β-adrenergic signaling plays a pivotal role in the modulation of cardiac function in physiological and pathological conditions. Understanding the molecular mechanism of β-adrenergic signaling is fundamental for heart disease therapy. A recent discovery of Rad, a novel endogenous regulator of LTCC, brought new insights into the βAR-cAMP-PKA signaling pathway, and well explained the controversial evidence on the functional phosphorylation sites on LTCC subunits. It is well known that β_1_AR mediates global cAMP signaling while β_2_AR generates localized cAMP signaling. Recent findings suggest that β_2_AR may also blunt β_1_AR signaling through GRK2 mediated “offside compartmentalization” mechanism, which can also serve as a negative feed-forward mechanism preventing the cell toxicity of tonic β_1_AR activation by circulating catecholamines. This underscores the critical protective role of β_2_AR against the detrimental effects of β_1_AR overstimulation. Further discoveries of βAR signaling mechanisms will contribute to novel and effective diagnostic and therapeutic heart disease targets.

## Conflict of interest

Bo Yang, Shi-Qiang Wang and Hua-Qian Yang declare that they have no conflict of interest.
